# SHORT-TERM ASSOCIATIONS OF ACTIVITY ENGAGEMENT WITH COGNITIVE PERFORMANCE

**DOI:** 10.1093/geroni/igae098.1884

**Published:** 2024-12-31

**Authors:** Minxia Luo, Charles Driver, Stacey Scott, Daisy Zavala, Charles Hall, Christina Roecke

**Affiliations:** University of Zurich, Zurich, Zurich, Switzerland; University of Zurich, Zurich, Zurich, Switzerland; Stony Brook University, Stony Brook, New York, United States; Stony Brook University, EAST SETAUKET, New York, United States; Albert Einstein College of Medicine, Bronx, New York, United States; University of Zurich, Zurich, Zurich, Switzerland

## Abstract

Prior research showed that activity engagement had short-term effects on numerical working memory over hours in German-speaking older adults. However, it is unclear whether these effects could be generalized to other cognitive outcomes and populations. This study examined short-term associations of activity engagement with cognitive performance using data from two studies that included adults of different demographic backgrounds in the US. That is, 16 days’ data from 282 older adults aged 70 to 90 years (M = 76.62, SD = 4.68; 66% women; 44% White, 40% Black, 11% Hispanic White, 3% Hispanic Black) from the Einstein Aging Study (EAS) and 14 days’ data from 260 participants aged 25 to 65 (M = 46.49, SD =11.09; 65% women; 8% White, 63% Black, 18% Hispanic White, 6% Hispanic Black) from the Effects of Stress on Cognitive Aging, Physiology, and Emotion Project (ESCAPE). For 4 or 5 times per day, participants reported whether they engaged in different social and cognitive activities (e.g., social interactions, TV watching) and completed smartphone cognitive assessments (i.e., processing speed, spatial working memory). Using hierarchical continuous-time dynamic modeling, we did not find any time-lagged associations between activity engagement and cognitive performance. Additionally, age, sex, and race-ethnicity did not moderate the time-lagged associations. Our results suggest that activity engagement did not have short-term effects on processing speed and spatial working memory in young and older populations with ethnic and racial diversity.

